# Targeting Glutathione Metabolism: Partner in Crime in Anticancer Therapy

**DOI:** 10.3390/nu11081926

**Published:** 2019-08-16

**Authors:** Enrico Desideri, Fabio Ciccarone, Maria Rosa Ciriolo

**Affiliations:** 1 Department of Biology, University of Rome “Tor Vergata”, Via della Ricerca Scientifica, 00133 Rome, Italy; 2 IRCCS San Raffaele Pisana, Department of Human Sciences and Promotion of the Quality of Life, San Raffaele Roma Open University, 00166 Rome, Italy; 3 IRCCS San Raffaele Pisana, Via della Pisana 235, 00163 Rome, Italy

**Keywords:** GSH, cancer therapy, ferroptosis, synthetic lethality

## Abstract

Glutathione (GSH) is the predominant low-molecular-weight antioxidant with a ubiquitous distribution inside the cell. The steady-state level of cellular GSH is dependent on the balance between synthesis, hydrolysis, recycling of glutathione disulphide (GSSG) as well as cellular extrusion of reduced, oxidized, or conjugated-forms. The augmented oxidative stress typical of cancer cells is accompanied by an increase of glutathione levels that confers them growth advantage and resistance to a number of chemotherapeutic agents. Targeting glutathione metabolism has been widely investigated for cancer treatment although GSH depletion as single therapeutic strategy has resulted largely ineffective if compared with combinatorial approaches. In this review, we circumstantiate the role of glutathione in tumour development and progression focusing on how interfering with different steps of glutathione metabolism can be exploited for therapeutic purposes. A dedicated section on synthetic lethal interactions with GSH modulators will highlight the promising option of harnessing glutathione metabolism for patient-directed therapy in cancer.

## 1. Introduction

The tripeptide, γ-l-glutamyl-l-cysteinyl-glycine, typically known as glutathione (GSH) is the most abundant low-molecular-weight thiol synthesized in the cell with a concentration in the low millimolar range (1–10 mM) in humans [1,2,3]. GSH shows ubiquitous distribution and most of the total GSH (about 90%) is stored in the cytosol. The remaining fraction of GSH is present in mitochondria, nucleus and endoplasmic reticulum [4]. Relatively low concentrations of GSH can be also found in the extracellular space [1,5]. GSH is an antioxidant involved in the scavenging of reactive oxygen/nitrogen species (ROS/RNS) and a detoxifying agent [2,3]. GSH is present in three main forms, reduced GSH, glutathione disulphide (GSSG) and glutathione-protein mixed disulphides (PSSG). Under physiological conditions, reduced GSH is the predominant form and it is 10 to 100 times more abundant than the oxidized form [1,2]. Together with Nicotinamide adenine dinucleotide phosphate (NADP/NADPH) and Thioredoxin (Trx(SH)_2_/TrxSS) systems, the GSH/GSSG redox couple determines the redox state in biological systems [2,6]. In addition to maintaining the intracellular redox potential, GSH has many other essential functions. Indeed, GSH stores most of the intracellular cysteine and modulates the activity of proteins via reversible protein glutathionylation, influencing cell cycle progression, cell death, transcription factor activity and signalling [1,2,4]. Reversible protein glutathionylation occurs non-enzymatically via thiol-disulphide exchange reactions between GSSG and a cysteinyl residue in a protein or via reaction of GSH with an activated thiol derivative such as sulfenic acid (–SOH), thiyl radical (–S⦁) and S-nitrosyl (–SNO) [4,7]. Due to the high intracellular GSH/GSSG ratio, thiol-disulphide exchange is an unlikely mechanism for protein glutathionylation even under severe oxidative stress. One exception is represented by c-Jun, which is susceptible to modification by GSSG at relatively high GSH/GSSG ratios [8]. Protein glutathionylation has also the function of protecting proteins from oxidative stress. Alteration of GSH homeostasis has a profound impact on cellular physiology and it has been demonstrated to be common in many pathological conditions including diabetes, neurodegenerative disorders and cancer [1,2,3]. In this review, we discuss the main alterations of GSH metabolism found in cancer and how GSH homeostasis can be manipulated or exploited for therapeutic purposes.

## 2. GSH Metabolism 

**Figure 1:**
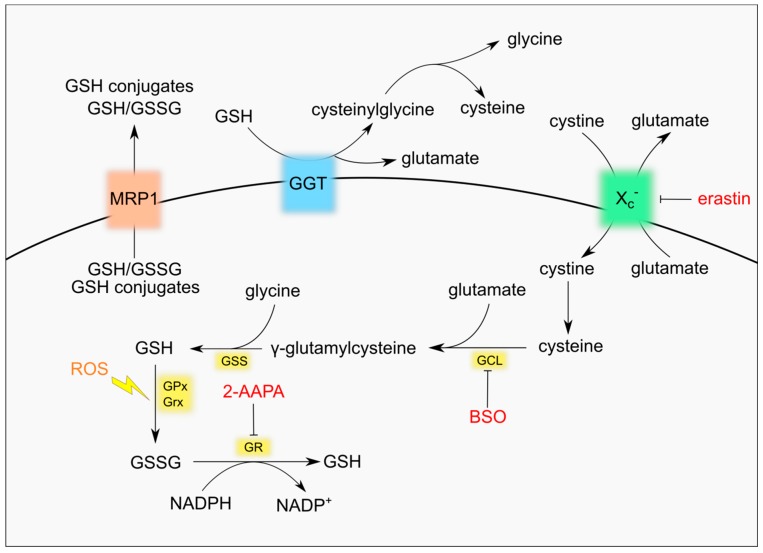
Glutathione (GSH) metabolism and druggable targets.

The synthesis of GSH takes place in the cytosol from its constituent amino acids cysteine, glutamate and glycine by two ATP-dependent enzymatic reactions. The first and limiting step is the conjugation of cysteine with glutamate to form the dipeptide γ-glutamylcysteine. The reaction is catalysed by γ-glutamyl-cysteine ligase (GCL), a heterodimeric enzyme composed of the catalytic subunit GCLc and the regulatory subunit GCLm. In the second reaction the enzyme glutathione synthetase (GSS) adds glycine to γ-glutamylcysteine and produces GSH [9] (Figure 1). The presence of the cysteine residue allows GSH to be oxidized to GSSG both non-enzymatically by free radicals or ROS/RNS and enzymatically by enzymes belonging to the family of glutathione peroxidases (GPxs) [3,10]. In fact, GSH antioxidant roles entail the direct reaction with superoxide anion radical [11] or the regeneration of non-enzymatic and enzymatic antioxidants, including α-tocopherol, which localizes to cell membrane where it prevents lipid peroxidation, and GPxs, that allow detoxification of lipid hydroperoxides and H_2_O_2_ [3,10,12]. Moreover, GSH is required for the activity of glutathione-S-transferases (GSTs), which are involved in the detoxification of xenobiotic substrates or products of oxidative stress by conjugation to GSH [13], and for glutaredoxin (GRx) catalysis, which reduces disulphide substrates, as well as PSSG, and produces GSSG [2,6]. Conditions of oxidative stress increase the conversion of GSH to GSSG. Accumulation of GSSG is potentially toxic for the cell, as it acts as a pro-oxidant, and it is promptly reduced back to GSH by glutathione reductase (GR) using NADPH, which is mainly produced by the oxidative branch of the pentose phosphate pathway (PPP) [2,3]. In response to excessive oxidative stress GSSG can be secreted from cells through specific transporters, contributing to a net decrease of intracellular GSH [14,15]. An active efflux of GSH has also been shown to occur in response to pro-apoptotic stimuli [16]. 

The presence of a γ-carboxyl group makes GSH resistant to the activity of most intracellular peptidases, increasing its stability within the cell [2,3,9]. GSH degradation in mammals was classically believed to occur only at the plasma membrane where the enzyme γ-glutamyl transpeptidase (GGT) localizes. The active site of GGT faces outside the cell, thus it can salvage GSSG or glutathione conjugates only when they efflux out of the cytosol via specific transporters. The GGT activity yields cysteinylglycine or bis-cysteinylglycine, which are cleaved by membrane-localized peptidases releasing cysteine (or cystine) and glycine, and glutamate obtained after the activity of glutamyl cyclotransferase (γ-GCT) and an ATP-dependent 5-oxoprolinase enzyme [2,17,18]. Specific transporters can bring back inside the cell all amino acids derived from GSH degradation. In particular, the glutamate/cystine antiporter system x_c_^−^ is essential for the uptake of cystine, the oxidized form of cysteine and the rate-limiting substrate for the de novo synthesis of GSH [19]. Recently, new enzymes have been shown to degrade GSH in the cytosol, expanding the complexity of GSH turnover. In fact, the Cation transport regulator homolog ChaC family of γ-GCTs starts glutathione degradation that is finally accomplished by 5-oxoprolinase that produces glutamate and by cysteinylglycine peptidases that produce cysteine and glycine [18,20,21].

## 3. GSH in Cancer

The contribution of glutathione to cancer is easy to guess if one considers the multiple roles that ROS have in tumour development and/or progression. Cancer cells exhibit elevated and persistent levels of oxidative stress as a consequence of the high metabolic rate and/or the activation of ROS-coupled signalling pathways. The electron transport chain represents the main endogenous source of ROS of either regular mitochondrial oxidative metabolism or dysfunctional mitochondria with low coupling efficiency and raised electron leakage. Moreover, many proliferative pathways are associated with the activation of membrane-bound NADPH oxidases which are the main source of ROS in signal transduction.

In the establishment of the tumour phenotype, if the high levels of ROS are not sufficiently buffered by antioxidant defence they can induce oxidative damage to DNA in terms of base damage, chromosome rearrangements, single- and double-strand breaks that can contribute to oncogenic activation or silencing of tumour suppressor genes [22,23]. Other oxidative damages that can raise the endogenous stress level in a cell include protein carbonylation and lipid peroxidation. Redox activation of signalling pathways involved in cell migration, survival and epithelial to mesenchymal transition was instead shown to contribute to cancer progression [24,25,26]. 

Based on this, glutathione and antioxidants in general can exhibit a dual role in cancer by safeguarding cell homeostasis from pro-neoplastic oxidative damages or by promoting cancer progression to avoid the activation of cell death pathways. The deregulation of glutathione metabolism is broadly identifiable in the majority of cancers as the genes involved in GSH turnover or utilization are under the transcriptional control of classical tumorigenic pathways, primarily the nuclear factor erythroid 2-related factor 2 (NRF2) signalling which drives the antioxidant response and control the transcription of GCL. For instance, in PI(3)K/Akt-driven breast cancer, NRF2 is stabilized and activated, promoting GSH biosynthesis and resistance to oxidative stress [27]. Moreover, NRF2 promotes GSH synthesis also from a metabolic point of view as demonstrated in tumours bearing mutated Kelch-like ECH-associated protein 1 (KEAP1), a negative regulator of NRF2 stability, in which glutamine-derived glutamate is used for GSH production at the expense of the Krebs cycle [28]. The hypoxia-inducible factor 1 (HIF1) pathway also activates GCL expression and GSH synthesis in hypoxic conditions and it was demonstrated to promote the enrichment of breast cancer stem cell niche following chemotherapy treatments [29]. In ovarian clear cell carcinoma, the hepatocyte nuclear factor-1β (HNF-1β), a transcription factor with important role in organogenesis and overexpressed in several cancer types [30], was shown to regulate GCL expression, extending the network of factors regulating glutathione synthesis outside those associated with oxidative stress [31]. Besides the de novo synthesis, another way exploited by tumour cells to increase GSH content is the upregulation of the PPP and thus NADPH useful for GR-mediated reduction of GSSG [32,33], which can be accumulated in tumour cells facing high levels of oxidative stress. 

Indirect evidence of GSH in decreasing cancer risk is given by the key role played in the reaction of GSTs for the detoxification of dangerous compounds, including carcinogens. Several polymorphisms of GSTs are known and they have been associated with increased incidence of virtually all tumour types. On the other side, many papers have shown that increased levels of glutathione actually promote cell cycle progression as also confirmed by studies performed on animal models. In fact, mutations of GCLm are able to delay the onset of sarcomas or lymphomas [34]. In humans, changes in the number of GAG triplet in the 5′-UTR of the GCLc gene were associated with an increased susceptibility to develop lung and aerodigestive tract cancers [35]. The role of GSS in cancer is currently less studied than GCL. Genetic variations of GSS have been associated with overall survival of small cell lung cancer patients and predict the recurrence in bladder cancer patients [36,37]. GGT, the enzyme responsible for the degradation of extruded GSH, is upregulated in several tumour types, favouring the uptake of GSH constituents, and its expression correlates with therapeutic resistance and poor prognosis in breast and renal cancer patients [38].

A key role of glutathione is also emerging in the context of tumour microenvironment. In particular, cancer-associated fibroblasts (CAFs) were shown to diminish the accumulation of genotoxic agents in cancer cells in a glutathione-dependent fashion. In fact, CAFs release high levels of thiols, including glutathione and cysteine, which increase intracellular GSH levels in tumours counteracting drug-dependent oxidative stress and apoptotic response [39,40].

## 4. Manipulation of GSH Homeostasis for Cancer Treatment

Many cancer types, including liver, lung, breast and colon cancers show elevated GSH levels with respect to normal tissues and take advantage of the detoxifying ability of GSH to counteract the activity of antineoplastic agents [41]. Therefore, GSH system drew the attention of researchers and several strategies aimed at reducing intracellular GSH have been developed in the attempt of blocking the growth of tumour cells and increasing the efficacy of existing anticancer therapies. 

### 4.1. Inhibition of GSH Synthesis 

The most straightforward strategy to decrease GSH level is the use of buthionine sulfoximine (BSO), the irreversible inhibitor of the enzyme catalysing the first and limiting step of GSH synthesis, GCL. Although not very efficient in reducing the growth of most cancer cells [42], BSO reduces the mammary tumour burden in MMTV-PyMT mice, which spontaneously develop breast cancer [34], and increases the efficacy of the commonly used anticancer agents, cisplatin and carboplatin [43,44]. BSO was also shown to increase susceptibility of breast cancer stem cells to nanoradiotherapy promoting ROS accumulation and apoptotic cell death [45]. Notably, GSH depletion by BSO induces ferroptosis, a recently identified non-apoptotic cell death mechanism characterized by increased lipid peroxidation [46]. Induction of ferroptosis may explain, at least in part, the synergistic effect of GSH depletion with anticancer drugs and may find application in circumventing the resistance to apoptosis typical of certain tumour cells [47]. In preclinical in vivo models BSO potentiates the efficacy of melphalan, an alkylating agent commonly used in the treatment of multiple myeloma [48]. The combination of BSO and melphalan underwent Phase I clinical trials in patients with different types of cancers and, more recently, in paediatric patients with recurrent/resistant high-risk neuroblastoma [49,50]. In the latter case, inhibition of GSH synthesis was able to increase the response to melphalan. Nevertheless, the short half-life of BSO [51], which would require prolonged infusions to maintain constant BSO blood levels, and the increased leukopenia and thrombocytopenia observed in some of the clinical studies may limit the use of BSO in a clinical setting. 

### 4.2. Reduction of GSH Precursors Availability

An alternative approach to decrease GSH levels is to reduce the availability of GSH precursors. An identified target is the glutamate/cystine antiporter system x_c_^−^, whose inhibition reduces the uptake of cystine and, as a consequence, GSH levels [19]. The x_c_^−^ inhibitor sulfasalazine was tested in a Phase I/II study for the treatment of progressive malignant gliomas but the study was early terminated due to lack of response and severe toxicity [52]. More recently another x_c_^−^ inhibitor, erastin, was shown to potentiate the effect of the apoptotic inducer tumour necrosis factor-related apoptosis-inducing ligand (TRAIL) in colon cancer cell lines [53]. Similarly to BSO, inhibition of cystine import by erastin activates ferroptosis, and it was hypothesized to contribute to the decreased radioresistance of lung cancer cells [53,54]. A Phase I clinical trial testing the safety of erastin analogue PRLX 93936 in patients with advanced solid tumours was completed in 2012 (NCT00528047) and a Phase I/II study started in the same year (NCT01695590). 

GSH precursors can be also obtained by the cells after degradation of extruded GSH by GGT. A number of inhibitors of GGT have been produced and tested, like acivicin and 6-diazo-5-oxol-norleucine (DON), facing the problem of toxicity which prevents their use in vivo [55].

### 4.3. Increase of GSSG Levels

GSH buffers oxidative stress produced by many anticancer drugs, resulting in the formation of GSSG. GSSG is rapidly reduced back to GSH by GR to prevent GSSG accumulation that would cause activation of redox-mediated cell death [56]. Increasing the intracellular concentration of GSSG has been hypothesized to be a good strategy to induce tumour cell death. Treatment with the irreversible GR inhibitor 2-AAPA sensitizes several cancer cell lines to X-Ray irradiation and is associated with a massive increase of GSSG and total disulphides [57]. GSSG reduction by GR requires NADPH from PPP. Inhibition of the first enzyme of PPP, glucose-6-phosphate dehydrogenase, increases oxidative stress, blocks the growth of head and neck squamous carcinoma cells in vivo [58] and re-sensitizes cisplatin-resistant cells [58]. A formulation of GSSG (NOV-002) was tested in combination with carboplatin/paclitaxel and showed promising results in Phase I and II clinical trials [59] but it eventually failed to increase overall survival in patients with advanced non-small cell lung cancer (NSCLC) in a Phase III study [60]. NOV-002 was also tested in combination with doxorubicin and cyclophosphamide in patients with breast cancer in a Phase II study, showing increased pathologic complete response rates [61]. 

### 4.4. Promotion of GSH Efflux 

Intracellular GSH homeostasis is maintained not only by the rate of GSH synthesis but also by its export via plasma membrane transporters [14]. Forcing the efflux of GSH can therefore decrease GSH levels and sensitize cancer cells to chemotherapy. The ATP binding cassette (ABC)-family transporter multidrug resistance protein 1 (MRP1) is upregulated in many tumour types and is associated with multidrug resistance [62]. It is also responsible for GSH efflux [63]. Cells expressing high levels of MRP1 are less sensitive to pro-apoptotic drugs but at the same time they are collaterally more sensitive to pro-ferroptotic agents, as recently shown by Dixon’s group [64]. We have shown that an increased efflux of GSH through MRP1 in carcinoma cell lines occurs in response to nutrient deprivation to modulate the activation of autophagy [65]. Caloric restriction and/or intermittent fasting are currently receiving great attention as promising schemes to enhance the effect of anticancer therapies, with encouraging results [66,67,68,69]. It would be interesting to verify whether this approach is particularly efficient against tumours with high levels of MRP1 that might lose more GSH than their untransformed counterparts and be more sensitive to oxidative stress.

## 5. Moving towards Precision Medicine: GSH in Synthetic Lethal Approaches

Cells bearing a tumour-specific mutation often become addicted to the activity of a partner gene to maintain their viability. Once identified, the synthetic lethal partner gene becomes an excellent therapeutic target to specifically kill cancer cells without affecting normal cells. The synthetic lethality concept was proposed more than 20 years ago but it received renewed attention with the coming of whole genome sequencing and mutational analysis [70]. The identification of tumour-specific mutations could indeed anticipate tumour vulnerabilities and thus responsiveness to a therapeutic regimen, paving the way for personalized targeted therapies. Several examples of synthetic lethal approaches involve DNA damage response pathways including non-homologous end joining (NHEJ) and homologous recombination (HR) for double-strand break (DSB) repair or base excision repair (BER) for single-strand break (SSB) damages. In fact, genome instability in tumours is frequently associated with mutations in genes involved in DNA damage response and activation of alternative DNA repair pathways as compensatory mechanisms to stressful/damaging insults. A renowned lethal interaction regards the inhibition of the BER pathway enzyme Poly (ADP-ribose) polymerase-1 (PARP-1) in ovarian and breast tumours bearing inactivating mutations in BRCA1/2 genes, which are involved in HR [71]. Other examples include the inhibition of Werner Syndrome (WRN) helicase (NHEJ/BER) in tumours with mutations in Fanconi anaemia-associated genes (interstrand cross-link repair) or the sensitivity of colorectal cancer cells deficient for the Bloom Syndrome (BLM) helicase (HR) to inhibition of the antioxidant enzyme SOD1 [72]. 

In 2003, a high-throughput synthetic lethality screening by Stockwell’s group identified a synthetic lethal interaction between cells expressing oncogenic Harvey rat sarcoma viral oncogene homolog (HRAS) and erastin [73]. About ten years later, the same group showed that erastin blocks the x_c_^−^ system and inhibits GSH synthesis, in one of the first examples of synthetic lethality connected to GSH metabolism [46]. Erastin efficiently kills also hereditary leiomyomatosis and renal cell cancer cells characterized by inactivation of the enzyme fumarate hydratase (FH), through induction of ferroptosis [74]. The acquisition of mutations driving oncogenic transformation confers an advantage to tumour cells but can also bring along collateral effects that expose vulnerabilities. This concept is well exemplified by the work of Okamoto’s group. Inactivation of the chromatin-remodelling factor AT-rich interactive domain-containing protein 1A (ARID1A) is found at high frequency in multiple cancer types and it is thought to promote tumorigenesis by interfering with DNA repair, like ROS-induced DSBs. A collateral effect of ARID1A inactivation is the decreased expression of the x_c_^−^ system, which makes cells lacking ARID1A sensitive to GCL inhibition, while untransformed cells are not [75]. Insufficient DNA damage response in ARID1A-deficient cancer cells is likely to further increase oxidative stress induced DSBs, ultimately leading to cell death. A similar phenomenon was observed in connection with p53. Indeed, cells that accumulate mutant p53 show reduced expression of the x_c_^-^ system and are particularly sensitive to inhibition of this transporter [76]. A relevant synthetic lethal interaction was also demonstrated for NRF2 pathway activation and glutaminase inhibition. In fact, glutamine anaplerosis was necessary for glutathione homeostasis in several tumors that exhibit KEAP1 mutation with consequent NRF2 constitutive activation [28,77], including the highly aggressive lung adenocarcinoma bearing co-mutations in Kirsten Rat Sarcoma Viral Oncogene Homolog (KRAS) and Liver Kinase B1 [78]. An interesting observation from Mak’s group led to the identification of another synthetic lethal interaction involving GSH. As mentioned before, BSO treatment increased ROS levels and reduced tumorigenesis in a mouse model of breast cancer but it was completely ineffective in established tumour [34]. Tumour cells upregulated and became dependent on the thioredoxin system, revealing a tumour vulnerability that can be efficiently targeted in a combination therapy approach. The synthetic lethality of GSH depletion and inhibition of thioredoxin system was demonstrated also in other studies [79,80]. However, combined inhibition of GSH synthesis and thioredoxin may fail to induce cancer cell death. Cells can indeed activate NRF2 as resistance mechanism. Many antioxidant enzymes are downstream target of NRF2 and can help to cope with the increased oxidative stress induced by GSH and Trx inhibition. A triple inhibition of GSH, Trx and NRF2 pathways was shown to overcome resistance and kill head and neck cancer cells [81]. While one could anticipate that combined inhibition of keys antioxidant systems would synergize and kill cancer cells, other reports highlighted additional mediators of resistance to GSH depletion that were more difficult to foresee. Taking advantage of publicly available data from genome-scale pooled CRISPR-Cas9 screens of more than 300 cancer cell lines, Harris et al. showed that most cancer cells are resistant to GSH depletion [42]. They also identified the deubiquitinating enzymes (DUBs) ubiquitin-specific protease 7 (USP7) and ubiquitin C-terminal hydrolase L5 (UCHL5) as novel mediators of resistance to GSH inhibition in two breast cancer cell lines. How DUBs mediate resistance to GSH depletion is still unknown but it is likely linked to reduced ubiquitination of oxidized proteins that provides an opportunity for protein refolding. In head and neck squamous cell carcinoma (HNSCC) cells resistance to GSH depletion was driven by upregulation of aldehyde dehydrogenase 3A1 (ALDH3A1) [82]. In fact, ALDH3A1 detoxifies lipid peroxides and the combined inhibition of ALDH3A1 and the x_c_^−^ system dramatically increase the levels of 4-hydroxynonenal, a highly reactive product of lipid peroxidation able to induce oxidative damage and cell death. All the identified mediators of resistance to GSH depletion become potential synthetic lethal therapeutic targets to selectively trigger cell death. Additional mechanisms driving resistance to GSH deficiency are likely existing but yet unknown. Their identification demands for additional efforts but will result in novel opportunities for targeted anticancer therapies. 

## 6. Conclusions

High GSH levels observed in many cancer cells allow the cells to cope with the oxidative stress caused by their increased metabolism and proliferation rate and protect them from the activity of chemotherapeutic agents. A lot of efforts have been put to target GSH metabolism with the aim of potentiating the efficacy of drugs causing oxidative stress or circumventing acquired resistance to chemotherapy. Nevertheless, the nonselective nature of thiol-depleting agents, including BSO, is a relevant adverse effect of GSH depletion strategies as they can irreversibly damage non-malignant tissues. Moreover, the development of mechanisms driving resistance to GSH deficiency, primarily enhancement of alternative antioxidant pathways mediated by Trx and NRF2 systems, is a challenge for pharmacological efficacy. In this context, another aspect to keep in mind when resistance to GSH depleting agents occurs is the intersection between GSH metabolism and most of cell death pathways, such as apoptosis, autophagy and necrosis [83].

To date, GCLc and the x_c_^−^ system are the preferential targets for the modulation of intracellular GSH in an anticancer perspective. Other potential targets have been discarded due to the high toxicity of inhibitors, which is the case of GGT, or never investigated with depth due to scarcity of potential inhibitors. A boost in the development of novel molecules may result from the identification of novel pathways regulating GSH metabolism, like the ChaC family of γ-GCTs. 

The coming of whole genome sequencing and high throughput screenings allowed the identification of synthetic lethal interactions with inhibition of GSH metabolism (Table 1). Indeed, although most cancer cells are resistant to GSH depletion, cells carrying specific genetic alterations are instead particularly sensitive. The identification of mutations which bring along sensitivity to GSH inhibition will open novel therapeutic windows and represent a further step towards the implementation of precision medicine approaches that take into account the unique characteristics of each person and that will eventually replace the one size fits all approach.

## Figures and Tables

**Table 1:** List of synthetic lethal interactions with inhibitors of Glutathione (GSH) metabolism.

Drug	Activity	Synthetic Lethal Partner	Effects	Cancer Model	Ref
**Erastin**	Inhibition of x_c_^−^ activity	HRAS^V12^	Increased lipid peroxidation/activation of ferroptosis	Engineered tumorigenic fibroblasts	[73]
		Accumulation of mutant p53		Oesophageal adenocarcinoma	[76]
		Inactivation of fumarate hydratase (FH)		Hereditary leiomyomatosis and renal cell cancer	[74]
**BSO Sulfasalazine**	Inhibition of GCLC Inhibition of x_c_^−^ activity	Inactivating mutation/ablation of ARID1A	Induction of ROS dependent apoptosis	Ovarian Cancer, endometrial carcinoma	[75]
**BPTES B-839**	Inhibition of glutaminase	Inactivating mutation of KEAP1	Suppression of cell growth	KRAS-mutant lung adenocarcinoma	[78]
**BSO**	Inhibition of GCLC	Inhibition of TrxR	Cancer cell death	Breast cancer, lung adenocarcinoma	[34]
		DUBs inhibition	Proteotoxic and ER stress-driven cell death	Breast cancer	[42]
**Sulfasalazine**	Inhibition of x_c_^−^ activity	Inhibitor of ALDH	Suppression of cell growth	Head and neck squamous cell carcinoma	[82]
